# Predictive value of neutrophil-to-apolipoprotein A1 ratio for early postoperative cerebral infarction in patients with ruptured cerebral aneurysms

**DOI:** 10.1186/s12944-026-02969-4

**Published:** 2026-05-14

**Authors:** Huangcheng Shangguan, Ye Xu, Yibin Zhang, Xiaojing Chen, Yi Wu, Yuanxiang Lin, Shufa Zheng, Peisen Yao, Xiaofen Huang, Dezhi Kang

**Affiliations:** 1 Department of Neurosurgery, Neurosurgery Research Institute, The First Affiliated Hospital, Fujian Medical University, Fuzhou, Fujian 350005 China; 2 Department of Neurosurgery, Binhai Campus of the First Affiliated Hospital, National Regional Medical Center, Fujian Medical University, Fuzhou, 350212 China; 3 Fujian Provincial Institutes of Brain Disorders and Brain Sciences, The First Affiliated Hospital, Fujian Medical University, Fuzhou, Fujian 350005 China; 4 Department of Medical Records Room, The First Affiliated Hospital, Fujian Medical University, Fuzhou, Fujian 350005 China

**Keywords:** Aneurysmal subarachnoid hemorrhage, Intracranial aneurysm, Cerebral infarction, Neutrophil, Inflammation, Apolipoprotein A1, Lipid metabolism

## Abstract

**Background and purpose:**

Early postoperative cerebral infarction (EPCI) remains a major complication following surgical treatment of aneurysmal subarachnoid hemorrhage (aSAH) and contributes to unfavorable neurological outcomes. The neutrophil-to-apolipoprotein A1 ratio (NAR), which indicates systemic inflammation and lipid-related vascular protection, has shown prognostic significance in multiple vascular diseases. However, its clinical value in identifying patients at risk of EPCI after aSAH remains unclear.

**Methods:**

This retrospective study included 517 patients with aSAH who underwent surgical treatment. Patients were divided into four groups according to the quartiles of their admission NAR. The relationship between NAR and EPCI was investigated using multivariable logistic regression, receiver operating characteristic curve analysis, restricted cubic spline modeling, subgroup analyses, and propensity score matching.

**Results:**

Of the 517 patients included, 90 (17.41%) developed EPCI. Higher NAR levels were associated with increased clinical and radiological severity, including higher Hunt–Hess and modified Fisher grades, as well as a significantly increased risk of EPCI. NAR was independently linked to EPCI after controlling for confounders (adjusted odds ratio = 1.16; 95% CI: 1.09–1.24; *P* < 0.001). aSAH patients in the highest quartile exhibited a 2.68-fold higher risk of EPCI compared to those in the lowest quartile. Receiver operating characteristic curve analysis indicated that NAR had moderate discriminative ability for EPCI, with an area under the curve of 0.698. Restricted cubic spline analysis demonstrated a positive dose-response association. These findings were consistent across subgroup analyses and were further corroborated by propensity score matching.

**Conclusions:**

Higher admission NAR independently correlated with an increased risk of post-operative EPCI and unfavourable prognosis. NAR, as an affordable and readily accessible biomarker, could offer practical benefits for early risk assessment and perioperative clinical management.

**Supplementary Information:**

The online version contains supplementary material available at 10.1186/s12944-026-02969-4.

## Introduction

 Aneurysmal subarachnoid hemorrhage (aSAH) is a devastating neurological emergency caused by intracranial aneurysm rupture, with a 30-day mortality of 40%–45%, and permanent neurological disability affecting approximately one-third of survivors [1–3]. Despite advances in microsurgical and endovascular therapies, cerebral infarction remains a common complication after aSAH, affecting approximately 21%–65% of patients and contributing substantially to morbidity and mortality [2]. Cerebral infarction following aSAH results from multiple mechanisms, including abrupt increases in intracranial pressure, neuroinflammation, microvascular thrombosis, and cortical spreading depolarizations, all of which impair cerebral autoregulation and promote irreversible neuronal injury [2, 4]. Early postoperative cerebral infarction (EPCI), which typically occurs within 72 h after aneurysm repair, substantially impairs neurological recovery and long-term quality of life [5–7]. Therefore, early recognition of patients at increased risk of EPCI is essential for timely intervention and improved outcomes.

Accumulating evidence indicates that systemic inflammation is a central contributor to secondary brain injury following aSAH. Neutrophils, as early effectors of innate immunity, infiltrate the central nervous system and contribute to brain injury through the production of reactive oxygen species (ROS) and proinflammatory mediators, thereby exacerbating vascular dysfunction and tissue injury [8–10]. Neutrophil-driven inflammation and disturbed lipid homeostasis have both been linked to the development of cerebral ischemia following aSAH [10, 11]. Apolipoprotein A1 (ApoA1), the main protein component of high-density lipoprotein, provides anti-inflammatory, antioxidative, and endothelial-protective benefits [12]. Reduced ApoA1 levels have been associated with impaired vascular integrity and adverse cerebrovascular outcomes [13].

The neutrophil-to-apolipoprotein A1 ratio (NAR) combines two biological processes, serving as a composite biomarker that indicates both inflammatory burden and lipid-mediated vascular protection. Elevated NAR has been reported to correlate with unfavourable outcomes in several conditions, such as ischemic stroke, heart failure, and malignancies [14–18]. Emerging evidence suggests that an elevated NAR may reflect a shift toward a proinflammatory state and diminished vascular protection, both of which may increase susceptibility to secondary ischemic events [14, 18].

However, the clinical significance of NAR in patients with aSAH has not been systematically assessed, particularly regarding its association with EPCI. It remains unclear whether any observed association is independent of established risk factors or if it follows a dose–response pattern. It was hypothesized that an elevated admission NAR would independently predict an increased risk of EPCI and might provide incremental value for early risk stratification. Accordingly, this study investigated the association of admission NAR with EPCI in aSAH patients.

## Materials and methods

### Study population

**Fig. 1:**
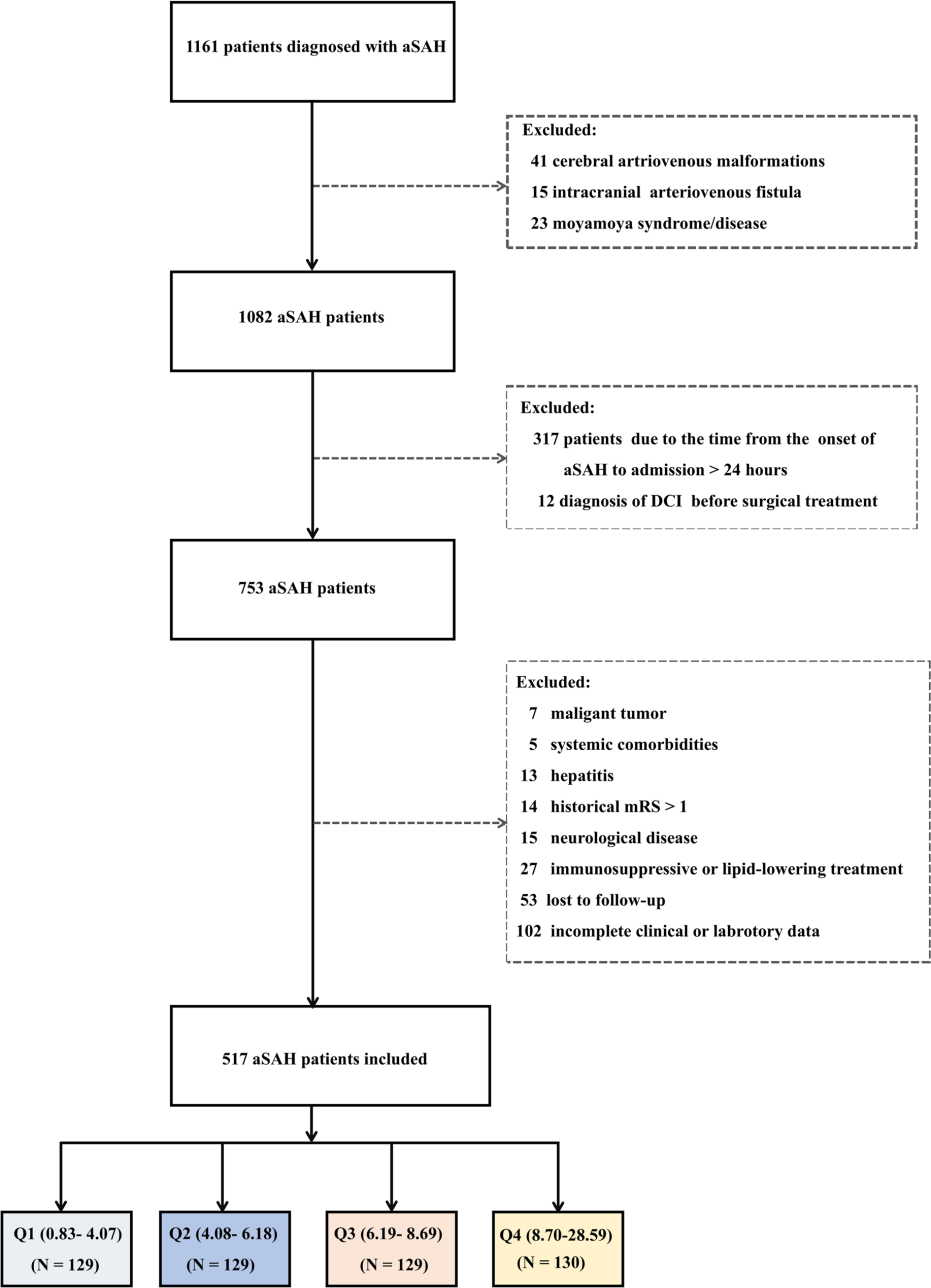
Patient selection flowchart

Consecutive patients with aSAH treated between January 2016 and December 2022 were retrospectively analysed. Ethical approval was granted by the Ethics Committee of the First Affiliated Hospital of Fujian Medical University (Approval No.: MRCTA, ECFAH of FMU [2022]601). Eligible patients were those age 18 years or older with a radiologically confirmed aSAH on computed tomography (CT), where the causative aneurysm was identified via CT angiography or digital subtraction angiography. They received surgical treatment within 72 h after symptom onset, had complete laboratory data obtained within 6 h of admission, and were admitted within 24 h after aSAH onset. Patients were excluded if they had (1) preexisting cerebral infarction or other significant cerebrovascular disease, (2) non-aneurysmal SAH, (3) severe hepatic or renal dysfunction or systemic inflammatory diseases, (4) death before imaging follow-up, (5) lipid-lowering or immunosuppressive treatment within 1 month before admission, (6) historical modified Rankin Scale (mRS) score > 1, (7) diagnosis of delayed cerebral ischemia (DCI) before surgical treatment, (8) loss to follow-up, or (9) missing key variables. Figure 1 illustrates the patient selection process.

### Clinical management of patients with aSAH after admission

After admission, all patients with aSAH were managed according to standardized institutional protocols. Initial management included airway protection, hemodynamic monitoring, neurological assessment, and intensive care monitoring. Supportive care measures involved adequate analgesia, maintaining normovolemia and strict blood pressure control [2]. Nimodipine was routinely administered for prophylaxis against DCI. External ventricular drainage (EVD) was performed in patients with hydrocephalus or elevated intracranial pressure when necessary. Definitive aneurysm exclusion was achieved at the earliest feasible time through microsurgical clipping or endovascular coiling, with treatment timing determined based on clinical status, aneurysm morphology, and logistical constraints.

### Data collection

Baseline variables extracted from electronic medical records included demographic characteristics, clinical presentation, aneurysm features, treatment modality, and admission laboratory parameters. Peripheral blood samples for laboratory analysis were obtained within 6 h of admission. Alcohol use was defined as drinking at least once per week for six consecutive months prior to admission, consistent with definitions used in large-scale Chinese epidemiologic studies on alcohol exposure [19]. Smoking status was defined as current or former smoking within the preceding 6 months. NAR was calculated as the absolute neutrophil count (×10^3^ cells/µL) divided by the ApoA1 level (mmol/L) [14–18].

$$NAR = \frac{Neutrophil\;count (10^3\; cells/ \mu L)}{ApoA1 (mmol/L)}$$

Clinical severity on admission was assessed using the Hunt-Hess grade (HHG) (I–V), with grades I–III categorized as mild and grades IV-V as severe [20]. Radiological severity was evaluated using the modified Fisher grade (mFG) based on admission CT, with grades 1–2 considered mild and grades 3–4 considered severe [20, 21]. Aneurysm characteristics included location, size, and neck width. Aneurysm locations were categorized into six groups according to established literature classifications [22, 23]. Aneurysm size was classified into four categories according to established clinical criteria : small (≤ 5 mm), medium (5.1–10 mm), large (10.1–25 mm), or giant (> 25 mm) [24, 25].

### Outcome definitionefinition

The primary outcome was EPCI, defined as a newly identified hypodense lesion on CT or a diffusion-restricted lesion on magnetic resonance imaging within 72 h after aneurysm repair, excluding infarcts attributable to surgical manipulation or ventricular catheter placement [3, 5, 7, 26]. All images were reviewed independently by two neuroradiologists blinded to clinical data. The secondary outcome was 3-month functional outcome, defined by the modified Rankin Scale (mRS) as favorable (0 to 2) or unfavorable (3 to 6).

### Statistical analysis

Continuous variables were assessed for distributional normality using the Shapiro-Wilk test. Normally distributed data are shown as mean ± standard deviation (SD), whereas skewed data are represented as median with interquartile range (IQR). Comparisons between two groups were made using the Student’s t-test or the Mann–Whitney U test for continuous variables, and the chi-square test or Fisher’s exact test for categorical variables.

Univariable analyses identified potential predictors of EPCI. Variables with *P <* 0.05 were entered into multivariable logistic regression models to determine independent predictors. NAR was assessed as both a continuous and categorical variable based on quartiles, with the lowest quartile (Q1) serving as the reference group. The crude model was unadjusted, whereas the adjusted model included variables significant in the univariable analysis.

Nonlinear associations between NAR and EPCI were explored using restricted cubic spline (RCS) analysis. The discriminatory ability of NAR was assessed with receiver operating characteristic (ROC) curve analysis, and the corresponding area under the curve (AUC) was calculated.

To minimize confounding, propensity score matching (PSM) was employed at a 1:2 ratio using nearest-neighbor matching without replacement. aSAH patients with and without EPCI were matched according to baseline characteristics. Subgroup analyses examined the consistency of the NAR-EPCI association across various strata, such as age, sex, hypertension, diabetes, smoking, alcohol use, and surgical modality. Interaction terms were added to the models to assess potential effect modification.

Statistical evaluations were performed with R software (v4.2.1), Empower (v2.0), SPSS Statistics (v26), and MedCalc (v18.2.1). *P* < 0.05 was deemed statistically significant.

## Results

### Baseline characteristics

Overall, 1,161 aSAH patients were initially screened, of whom 517 met the inclusion criteria. Among them, 90 (17.41%) developed EPCI. The Shapiro–Wilk test indicated that all continuous variables, except for total cholesterol, were non-normally distributed. Patients were categorized into quartiles according to admission NAR levels: Q1 (0.83–4.07), Q2 (4.08–6.18), Q3 (6.19–8.69), and Q4 (8.70–28.59). Baseline characteristics stratified by NAR quartiles are shown in Table 1. Notably, the incidence of EPCI increased progressively across NAR quartiles, from 8.53% in Q1 to 11.63% in Q2, 16.28% in Q3, and 33.08% in Q4. Higher NAR levels were associated with younger age, poorer clinical status (higher HHG), more severe SAH (higher mFG), AcomA aneurysms, increased EPCI incidence, and worse outcomes.

**Table 1:** Baseline characteristics stratified by NAR quartiles

Characteristics	Q1 (0.83–4.07)(N = 129)	Q2 (4.08–6.18)(N = 129)	Q3 (6.19–8.69)(N = 129)	Q4 (8.70–28.59)(N = 130)	P
Age, yrs, median (IQR)	57.00 (52.00–63.00)	55.00 (49.00–64.00)	54.00 (46.00–61.00)	52.00 (46.00–60.00)	0.001
Sex, n (%)					0.217
Male	43 (33.33)	51 (39.53)	51 (39.53)	60 (46.15)	
Female	86 (66.67)	78 (60.47)	78 (60.47)	70 (53.85)	
Hypertension, n (%)					0.100
No	71 (55.04)	56 (43.41)	52 (40.31)	60 (46.15)	
Yes	58 (44.96)	73 (56.59)	77 (59.69)	70 (53.85)	
Diabetes mellitus, n (%)					0.966
No	120 (93.02)	121 (93.80)	122 (94.57)	122 (93.85)	
Yes	9 (6.98)	8 (6.20)	7 (5.43)	8 (6.15)	
Smoking status, n (%)					0.645
No	115 (89.15)	111 (86.05)	108 (83.72)	113 (86.92)	
Yes	14 (10.85)	18 (13.95)	21 (16.28)	17 (13.08)	
Alcohol use, n (%)					0.044
No	115 (89.15)	105 (81.40)	114 (88.37)	102 (78.46)	
Yes	14 (10.85)	24 (18.60)	15 (11.63)	28 (21.54)	
Interval from symptom onset to initial CT (hours), median (IQR)	15.00 (11.00–17.00)	15.00(11.00–18.00)	14.00(8.00–19.00)	13.00(10.00–17.00)	0.405
HHG, n (%)					<0.001
Ⅰ–Ⅲ	114 (88.37)	118 (91.47)	98 (75.97)	81 (62.31)	
Ⅳ–Ⅴ	15 (11.63)	11 (8.53)	31 (24.03)	49 (37.69)	
mFG, n (%)					<0.001
1–2	102 (79.07)	100 (77.52)	88 (68.22)	70 (53.85)	
3–4	27 (20.93)	29 (22.48)	41 (31.78)	60 (46.15)	
Aneurysm characteristics, n (%)					<0.001
ACA	8 (6.20)	5 (3.88)	7 (5.43)	8 (6.15)	
AcomA	34 (26.36)	39 (30.23)	52 (40.31)	55 (42.31)	
ICA	34 (26.36)	23 (17.83)	6 (4.65)	9 (6.92)	
MCA	17 (13.18)	28 (21.71)	39 (30.23)	28 (21.54)	
PcomA	29 (22.48)	33 (25.58)	21 (16.28)	25 (19.23)	
Others	7 (5.43)	1 (0.78)	4 (3.10)	5 (3.85)	
Aneurysm size, n (%)					0.667
Small	56 (43.41)	53 (41.09)	59 (45.74)	63 (48.46)	
Medium	58 (44.96)	66 (51.16)	63 (48.84)	60 (46.15)	
Large	13 (10.08)	8 (6.20)	6 (4.65)	5 (3.85)	
Giant	2 (1.55)	2 (1.55)	1 (0.78)	2 (1.54)	
Aneurysm neck width, mm, median (IQR)	4.00 (2.80–5.00)	4.00 (3.00–5.00)	3.70 (2.80–5.00)	4.00(2.92–5.00)	0.897
Admission laboratory					
Hb (g/L), median (IQR)	131.00 (118.00–144.00)	129.00 (117.00–140.00)	128.00 (116.00–142.00)	132.50 (120.25–143.00)	0.150
Hct, median (IQR)	0.38 (0.35–0.42)	0.39 (0.34–0.42)	0.37 (0.34–0.42)	0.39 (0.36–0.42)	0.241
Platelet, ×10^9^/L, median (IQR)	209.00 (173.00–256.00)	200.00 (175.00–248.00)	216.00 (179.00–253.00)	233.00 (198.50–274.75)	0.002
Neutrophils, ×10^12^/L, median (IQR)	4.06 (2.90–4.81)	7.00 (6.00–8.20)	9.45 (8.14–10.82)	13.23 (11.09–15.67)	<0.001
HDL, mmol/L, median (IQR)	1.32 (1.13–1.74)	1.45 (1.18–1.69)	1.28 (1.07–1.52)	1.20 (1.02–1.43)	<0.001
LDL, mmol/L, median (IQR)	2.84 (2.22–3.45)	2.81 (2.36–3.49)	2.69 (2.12–3.38)	2.49 (1.78–3.18)	0.010
ApoB, mmol/L, median (IQR)	0.94 (0.77–1.14)	0.95 (0.81–1.15)	0.93 (0.74–1.12)	0.88 (0.68–1.09)	0.057
Triglyceride, mmol/L, median (IQR)	1.12 (0.78–1.69)	0.91 (0.69–1.22)	0.98 (0.73–1.38)	0.92 (0.54–1.43)	0.012
Total cholesterol, mmol/L,mean ± SD	4.82 ± 1.11	4.68 ± 1.20	4.42 ± 1.00	4.26 ± 1.39	<0.001
PT, seconds, median (IQR)	12.20 (11.50–12.80)	12.20 (11.60–12.90)	12.20 (11.40–12.90)	12.35 (11.90–13.10)	0.359
aPTT, seconds, median (IQR)	31.70 (27.70–35.50)	28.50 (24.90–33.20)	28.20 (24.00–33.10)	27.30 (23.52–33.18)	<0.001
Fibrinogen, g/L, median (IQR)	2.86 (2.40–3.93)	2.81 (2.20–3.73)	2.89 (2.37–3.69)	2.80 (2.20–3.63)	0.529
Surgical methods, n (%)					<0.001
Clipping	63 (48.84)	87 (67.44)	94 (72.87)	88 (67.69)	
Coiling	66 (51.16)	42 (32.56)	35 (27.13)	42 (32.31)	
EPCI, n (%)					<0.001
No	118 (91.47)	114 (88.37)	108 (83.72)	87 (66.92)	
Yes	11 (8.53)	15 (11.63)	21 (16.28)	43 (33.08)	
mRS, n (%)					<0.001
0–2	117 (90.70)	112 (86.82)	104 (80.62)	94 (72.31)	
3–6	12 (9.30)	17 (13.18)	25 (19.38)	36 (27.69)	

### Association between NAR and EPCI

Clinical, demographic, and laboratory factors associated with EPCI are presented in Table 2. Significant associations with EPCI were found for history of hypertension, HHG, mFG, neutrophils, high-density lipoprotein cholesterol (HDL-C), low-density lipoprotein cholesterol (LDL-C), ApoA1, and NAR (Table 2). Patients with EPCI exhibited a significantly higher incidence of unfavorable 3-month functional outcome compared to those without EPCI (mRS 3–6: 38.89% versus 12.88%, *P* < 0.001).

**Table 2:** Clinical, demographic, and laboratory factors associated with EPCI

Characteristics	Pre-PSM			Post-PSM		
	Non-EPCI(N = 427)	EPCI(N = 90)	P	Non-EPCI(N = 151)	EPCI(N = 85)	P
Age, yrs, median (IQR)	55.00 (48.00–62.00)	55.50 (48.25–63.75)	0.830	55.00 (47.00–63.50)	55.00 (48.00–63.00)	0.716
Sex, n (%)			0.134			0.413
Male	163 (38.17)	42 (46.67)		61 (40.40)	39 (45.88)	
Female	264 (61.83)	48 (53.33)		90 (59.60)	46 (54.12)	
Hypertension, n (%)			0.007			0.794
No	209 (48.95)	30 (33.33)		49 (32.45)	29 (34.12)	
Yes	218 (51.05)	60 (66.67)		102 (67.55)	56 (65.88)	
Diabetes mellitus, n (%)			0.450			0.436
No	399 (93.44)	86 (95.56)		140 (92.72)	81 (95.29)	
Yes	28 (6.56)	4 (4.44)		11 (7.28)	4 (4.71)	
Smoking status, n (%)			0.280			0.999
No	366 (85.71)	81 (90.00)		135 (89.40)	76 (89.41)	
Yes	61 (14.29)	9 (10.00)		16 (10.60)	9 (10.59)	
Alcohol, n (%)			0.725			0.611
No	359 (84.07)	77 (85.56)		124 (82.12)	72 (84.71)	
Yes	68 (15.93)	13 (14.44)		27 (17.88)	13 (15.29)	
Interval from symptom onset to initial CT (hours), median (IQR)	14.00(10.00–18.00)	15.00 (8.25–18.00)	0.730	14.00(9.00–18.00)	15.00 (9.00–18.00)	0.719
HHG, n (%)			<0.001			0.531
Ⅰ–Ⅲ	356 (83.37)	55 (61.11)		102 (67.55)	54 (63.53)	
Ⅳ–Ⅴ	71 (16.63)	35 (38.89)		49 (32.45)	31 (36.47)	
mFG, n (%)			<0.001			0.970
1–2	318 (74.47)	42 (46.67)		75 (49.67)	42 (49.41)	
3–4	109 (25.53)	48 (53.33)		76 (50.33)	43 (50.59)	
Aneurysm characteristics, n (%)			0.288			0.992
ACA	26 (6.09)	2 (2.22)		2 (1.32)	2 (2.35)	
AcomA	141 (33.02)	39 (43.33)		61 (40.40)	35 (41.18)	
ICA	63 (14.75)	9 (10.00)		16 (10.60)	9 (10.59)	
MCA	93 (21.78)	19 (21.11)		33 (21.85)	19 (22.35)	
PcomA	91 (21.31)	17 (18.89)		32 (21.19)	16 (18.82)	
Others	13 (3.04)	4 (4.44)		7 (4.64)	4 (4.71)	
Aneurysm size, n (%)			0.672			0.790
Small	193 (45.20)	38 (42.22)		72 (47.68)	36 (42.35)	
Medium	201 (47.07)	46 (51.11)		72 (47.68)	43 (50.59)	
Large	28 (6.56)	4 (4.44)		5 (3.31)	4 (4.71)	
Giant	5 (1.17)	2 (2.22)		2 (1.32)	2 (2.35)	
Aneurysm neck width, mm, median (IQR)	4.00 (2.80–5.00)	3.65 (2.92–5.00)	0.951	4.00 (2.75–5.00)	3.50 (2.90–5.00)	0.747
Admission laboratory						
Hb, (g/L), median (IQR)	129.00(117.00–141.00)	131.50(119.25–143.75)	0.184	129.00(117.00–141.00)	131.00(119.00–143.00)	0.344
Hct, median (IQR)	0.38 (0.35–0.42)	0.39 (0.36–0.42)	0.343	0.38 (0.35–0.42)	0.39 (0.36–0.41)	0.536
Platelet, ×10^9^/L, median (IQR)	216.00(178.00–256.00)	229.50(182.00–275.50)	0.080	226.00(184.00–272.50)	229.00(182.00–274.00)	0.994
Neutrophils, ×10^12^/L, median (IQR)	7.67 (5.01–10.32)	10.21 (6.73–13.57)	<0.001	8.86 (5.61–11.55)	10.30 (6.62–13.60)	0.021
HDL-C,mmol/L, median (IQR)	1.34 (1.11–1.59)	1.19 (0.96–1.54)	0.025	1.28 (1.10–1.52)	1.20 (0.96–1.54)	0.332
LDL-C,mmol/L, median (IQR)	2.80 (2.17–3.42)	2.46 (1.75–3.15)	0.004	2.57 (1.94–3.24)	2.48 (1.81–3.19)	0.588
ApoA1, mmol/L, median (IQR)	1.32 (1.16–1.52)	1.23 (0.93–1.39)	<0.001	1.28 (1.14–1.48)	1.24 (0.92–1.40)	0.031
ApoB, mmol/L, median (IQR)	0.94 (0.76–1.15)	0.84 (0.69–1.11)	0.037	0.89 (0.72–1.08)	0.87 (0.71–1.11)	0.696
Triglyceride, mmol/L, median (IQR)	0.96 (0.69–1.37)	1.06 (0.69–1.52)	0.608	1.00 (0.70–1.40)	1.06 (0.69–1.53)	0.834
Total cholesterol，mmol/L, mean ± SD	4.61 ± 1.16	4.25 ± 1.37	0.010	4.31 ± 1.06	4.29 ± 1.39	0.895
NAR, median (IQR)	5.84 (3.70–8.26)	8.46 (5.98–11.69)	<0.001	6.80 (4.62–9.21)	8.29 (5.97–11.62)	0.002
NAR, n (%)			<0.001			0.043
Q1	118 (27.63)	11 (12.22)		43 (28.48)	16 (18.82)	
Q2	114 (26.70)	15 (16.67)		40 (26.49)	19 (22.35)	
Q3	108 (25.29)	21 (23.33)		39 (25.83)	20 (23.53)	
Q4	87 (20.37)	43 (47.78)		29 (19.21)	30 (35.29)	
PT, seconds, median (IQR)	12.20 (11.60–13.00)	12.25 (11.60–12.90)	0.948	12.20 (11.55–12.80)	12.20 (11.60–12.90)	0.820
aPTT, seconds, median (IQR)	29.70 (24.90–33.95)	27.15 (24.10–31.93)	0.028	27.90 (24.00–33.30)	27.10 (23.90–32.00)	0.579
Fibrinogen, g/L, median (IQR)	2.83 (2.29–3.74)	2.82 (2.24–3.68)	0.727	2.83 (2.29–4.04)	2.81 (2.23–3.49)	0.488
Surgical methods, n (%)			0.133			0.866
Clipping	268 (62.76)	64 (71.11)		105 (69.54)	60 (70.59)	
Coiling	159 (37.24)	26 (28.89)		46 (30.46)	25 (29.41)	
mRS, n (%)			<0.001			0.002
0-2	372 (87.12)	55 (61.11)		122 (80.79)	53 (62.35)	
3-6	55 (12.88)	35 (38.89)		29 (19.21)	32 (37.65)	

**Fig. 2:**
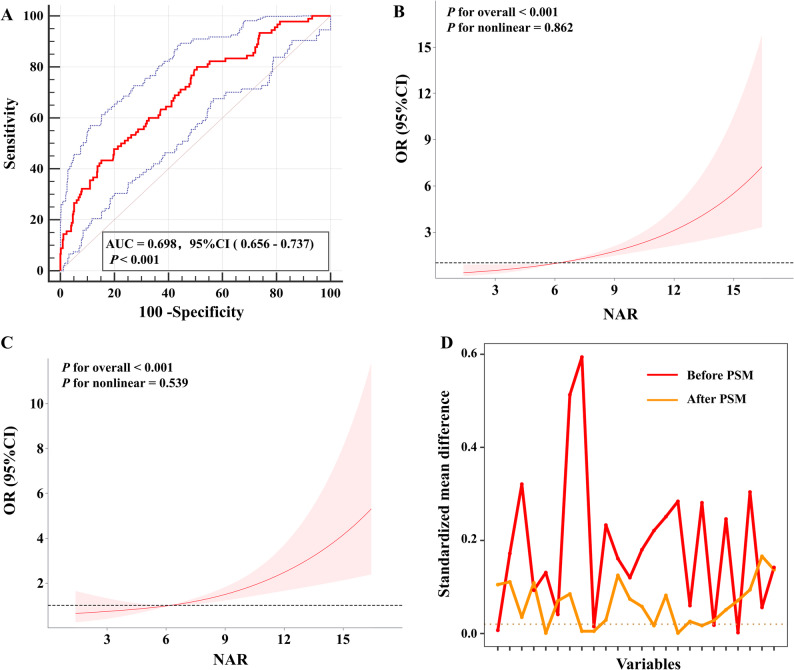
Association between NAR and EPCI. **A** ROC curve analysis of NAR for predicting EPCI. **B** Association between NAR and EPCI in the crude model. **C** Association of NAR with EPCI in the adjusted model after controlling for hypertension, HHG, mFG, ApoB, HDL-C, LDL-C, total cholesterol, PLT, and aPTT. The x-axis denotes NAR, while the y-axis indicates the relative likelihood of EPCI occurrence. The solid curve corresponds to the estimated OR, and the light shaded regions represent the 95% confidence intervals. **D** Standardized mean differences in covariates before and after PSM

A multivariate logistic regression model was employed to account for potential confounders, including covariates identified as significant in the univariate analyses. When NAR was modelled as a continuous variable, each unit increase was associated with higher odds of EPCI in both the crude model (odds ratio [OR] = 1.22, 95% confidence interval [CI]: 1.15–1.30, *P* < 0.001; Table 3), and the adjusted model (adjusted OR = 1.16, 95% CI: 1.09–1.24, *P* < 0.001; Table 3) after adjusting for hypertension, HHG, mFG, apolipoprotein B(ApoB), HDL-C, LDL-C, total cholesterol, platelets (PLT), and activated partial thromboplastin time (aPTT). Although the effect estimate was attenuated after adjustment, the association remained statistically significant, indicating that NAR contributed modest but independent information beyond established clinical and lipid-related variables. When analysed by quartile, the highest NAR quartile (Q4) was associated with greater odds of EPCI than the lowest quartile in both crude and adjusted models, with an adjusted OR of 2.68 (95% CI: 1.22–5.90, *P* = 0.014; Table 3). A significant trend across quartiles was also observed (trend *P* = 0.007). ROC curve analysis indicated that NAR had moderate discriminative performance for EPCI, achieving an AUC of 0.698 (95% CI: 0.656–0.737, *P* < 0.001; Fig. 2A).

**Table 3:** Estimated odds of EPCI according to admission NAR

Admission NAR	Crude OR (95% CI)	*P*	Adjusted OR (95% CI)	*P*
Per-unit increase	1.22 (1.15–1.30)	< 0.001	1.16 (1.09–1.24)	< 0.001
Quartile NAR				
Q1	Ref.		Ref.	
Q2	1.41 (0.62–3.20)	0.410	1.30 (0.55–3.07)	0.543
Q3	2.09 (0.96–4.53)	0.063	1.35 (0.59–3.09)	0.474
Q4	5.30 (2.59–10.87)	< 0.001	2.68 (1.22–5.90)	0.014
Trend *P*	< 0.001		0.007	

Crude model, no covariates adjustment

Adjusted model, adjusted for hypertension, HHG, mFG, ApoB, HDL-C, LDL-C, total cholesterol, PLT, and aPTT

### Dose–response and subgroup analysis

As shown in Fig. 2B, C, RCS analysis indicated a significant association between NAR and EPCI risk in both crude and adjusted models (both *P* < 0.001), without significant nonlinearity (*P* for nonlinearity = 0.862 and 0.539, respectively). EPCI risk increased noticeably at NAR values above approximately 6.0.

**Fig. 3:**
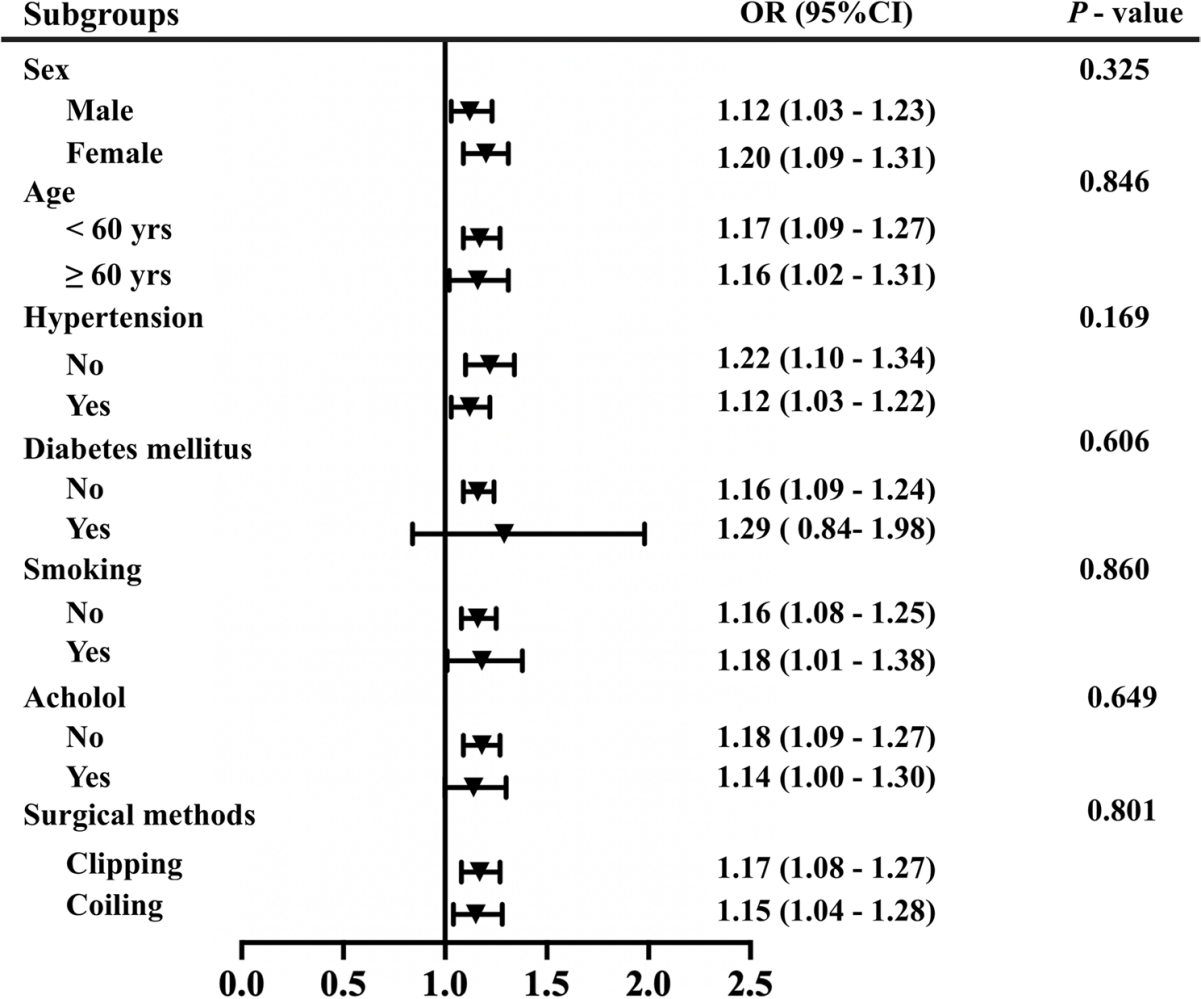
Association between NAR and EPCI across subgroups

Subgroup analyses showed a broadly consistent association between NAR and EPCI across strata defined by age (< 60 versus ≥ 60 years), sex, hypertension, diabetes, smoking, alcohol use, and treatment modality, with no evidence of interaction (all *P* for interaction > 0.05) (Fig. 3).

Furthermore, a 1:2 nearest-neighbor PSM analysis was performed to mitigate the potential confounding factors, yielding a matched cohort of 85 patients with EPCI and 151 patients without EPCI. Table 2 shows that post-matching, the baseline characteristics of the two groups were comparable (all *P* > 0.05). The adequacy of matching was further confirmed by standardized mean differences before and after matching, which demonstrated satisfactory covariate balance (Fig. 2D). After matching, higher NAR levels were still observed in the EPCI group (*P* < 0.05; Table 2), regardless of whether NAR was analysed continuously or by quartiles, in line with pre-matching results. Moreover, patients with EPCI exhibited significantly worse 3-month functional outcomes than those without EPCI (unfavorable outcome: 37.65% versus 19.21%, *P* = 0.002). Taken together, these findings support the robustness of the association between NAR and EPCI and further underscore its clinical relevance.

## Discussion

This study shows that higher admission NAR independently correlates with a greater risk of EPCI in aSAH patients. The association persisted after adjustment for confounders and remained stable in the PSM analysis. In addition, elevated NAR was linked to unfavorable 3-month functional outcomes. These findings suggest that NAR, an easily accessible biomarker, could be a useful tool for identifying patients at increased risk of early ischemic complications following aSAH.

Multivariable logistic regression showed that the association between NAR and EPCI remained statistically significant after adjustment for multiple confounders, with an adjusted OR of 1.16 (95% CI: 1.09–1.24). Although the effect size was modest, the association remained statistically significant, indicating that NAR is independently associated with EPCI risk. The attenuation of OR after adjustment was expected because key covariates, such as HHG, mFG, and lipid-related parameters, have strong effects on postoperative ischemic events. Thus, NAR should be interpreted as a supportive biomarker associated with EPCI risk and potentially useful for early risk stratification, rather than as a definitively validated additive predictor beyond established clinical models.

Previous studies have emphasized the critical role of systemic inflammation and disrupted lipid metabolism in driving secondary brain injury after aSAH [27–29]. Neutrophils, as early responders of the innate immune system, contribute to endothelial damage, facilitate microthrombus formation, and compromise cerebral perfusion [8, 9, 11, 23, 30]. In contrast, ApoA1, the principal protein component of HDL-C, exhibits anti-inflammatory, antioxidative, and endothelium-protective effects [12, 31]. An elevated NAR likely reflects a biological imbalance characterized by heightened inflammation and diminished vascular repair capacity, providing a plausible mechanistic explanation for its association with early ischemic events after aneurysm treatment. In this study, RCS analysis identified a nonlinear correlation between NAR and EPCI risk, with a notable inflection point at approximately 6.0. Even moderate increases in NAR were linked to a disproportionate rise in postoperative ischemic risk, underscoring the potential utility of early inflammatory-lipid assessment in patients with aSAH.

This observation is biologically plausible in light of prior evidence linking heightened neutrophilic inflammation and impaired HDL-C/ApoA1–mediated vascular protection to adverse outcomes after aSAH [11, 32–34]. However, this study extends that evidence by focusing specifically on NAR, a composite index that integrates inflammatory activation with lipid-related protective capacity. Compared with single biomarkers, NAR may better capture the complex interplay between harmful inflammatory responses and impaired vascular protection during the early critical phase after aSAH. In addition, the study investigated the predictive value of NAR for early postoperative infarction rather than DCI or vasospasm, which have more direct clinical relevance to surgical and perioperative management.

Various mechanisms could account for the link between increased NAR and EPCI. First, neutrophil activation may induce vascular endothelial dysfunction through the release of proteases, ROS, and proinflammatory cytokines, thereby promoting early microvascular thrombosis and no-reflow phenomena after aneurysm repair [8, 10, 11, 33, 35]. Second, neutrophil extracellular traps generated during excessive neutrophil activation can directly damage the vascular wall and contribute to a hypercoagulable state [9, 11, 35]. Third, reduced ApoA1 levels may weaken HDL-mediated anti-inflammatory and antioxidative effects, thereby exacerbating endothelial injury and microcirculatory failure [28, 32, 36]. Together, these processes may create a vulnerable environment that predisposes patients to early cerebral infarction after surgical or endovascular intervention.

### Strengths

This study is the first to assess the link between admission NAR and EPCI risk in aSAH patients. It specifically focused on EPCI occurring within 72 h after aneurysm treatment, a clinically relevant time window for perioperative management. NAR, being an accessible, cost-effective, and non-invasive biomarker, could effectively supplement existing clinical and radiological predictors. In addition, the use of multivariable adjustment and PSM strengthened the robustness of the findings.

### Limitations

Some limitations warrant consideration. First, the study’s retrospective, single-center design might have introduced selection bias and precluded causal inference. Thus, the relationship between NAR and EPCI should not be interpreted causally. Second, only a single baseline NAR measurement was analysed, preventing assessment of dynamic changes during hospitalization. Additionally, variability in laboratory sampling times and unrecorded perioperative factors, such as intraoperative complications, might have influenced the outcomes. Third, the analysis focused on early infarction within 72 h after aneurysm repair and did not assess DCI, a key determinant of long-term outcomes. Fourth, although systemic inflammatory diseases and recent lipid-lowering therapy were excluded and confounders were adjusted for, residual confounding remains possible. Fifth, while NAR was independently associated with EPCI, the added predictive value of NAR beyond existing clinical and radiological models was not formally evaluated. Therefore, the incremental predictive value of NAR beyond existing clinical models cannot be determined from the present study. Sixth, the lack of EVD data limited the assessment of its impact on cerebral perfusion and infarction risk. Finally, the absence of external validation and multicenter data restricted generalizability. Future studies should incorporate serial biomarker monitoring, perioperative EVD and intracranial pressure data, and long-term outcome evaluations to confirm and expand these findings.

## Conclusion

An increased NAR at admission independently correlated with a higher risk of EPCI and unfavorable functional outcomes in aSAH patients. NAR, being an affordable and easily accessible biomarker, might aid in early risk assessment upon hospital admission. Additional prospective multicenter studies are needed to validate these findings and assess whether NAR provides prognostic insights beyond existing clinical models.

## Supplementary Information

Supplementary Material 1

Supplementary Material 2

## Data Availability

All data obtained in this study are included in the paper and/or supplementary materials. Other data supporting the conclusions are available from the corresponding author upon request.

## Abbreviations

ACA: Anterior cerebral artery
AcomA: Anterior communicating artery
aPTT: Activated partial thromboplastin time
ApoA1: Apolipoprotein A1
ApoB: Apolipoprotein B
aSAH: Aneurysmal subarachnoid hemorrhage
AUC: Area under the curve
CI: Confidence interval
CT: Computed tomography
EPCI: Early postoperative cerebral infarction
Hb: Hemoglobin
Hct: Hematocrit
HHG: Hunt-Hess grade
ICA: Internal carotid artery
IQR: Interquartile range
MCA: Middle cerebral artery
mFG: Modified Fisher grade
mRS: Modified Rankin Scale
NAR: Neutrophil-to- apolipoprotein A1 ratio
OR: Odds ratio
PcomA: Posterior communicating artery
PT: Prothrombin time
SD: Standard deviation
