# Supplementary material for: Predictive value of neutrophil-to-apolipoprotein A1 ratio for early postoperative cerebral infarction in patients with ruptured cerebral aneurysms
Source: Lipids Health Dis. 2026 May 14;25:141. doi: 10.1186/s12944-026-02969-4 (PMC13217639; doi:10.1186/s12944-026-02969-4)
Supplement: Supplementary file 1 — Supplementary Material 1 [file 12944_2026_2969_MOESM1_ESM.pdf]

EDITORIAL CERTIFICATE

This certificate is issued as a confirmation that the paper mentioned below has been proofread and corrected by the HOME for Researchers' editorial team. After being checked and amended as seen appropriate, we feel that the standard of English in this manuscript satisfies the requirements of submission to journals to be considered for publication.

Manuscript Title

《Predictive Value of Neutrophil-to-Apolipoprotein A1 Ratio for Early Postoperative Cerebral Infarction in Patients with Ruptured Cerebral Aneurysms》

Certificate Number

20260000247

Date Issued

2026-04-08

HOME for Researchers
